# Native reptiles alter their foraging in the presence of the olfactory cues of invasive mammalian predators

**DOI:** 10.1098/rsos.180136

**Published:** 2018-10-31

**Authors:** C. Webster, M. Massaro, D. R. Michael, D. Bambrick, J. L. Riley, D. G. Nimmo

**Affiliations:** 1 Institute for Land, Water and Society, School of Environmental Sciences, Charles Sturt University, Albury, New South Wales 2640, Australia; 2 Fenner School of Environment and Society, The Australian National University, Canberra, Australian Capital Territory 2611, Australia; 3 Department of Biological Sciences, Macquarie University, Sydney, New South Wales 2109, Australia

**Keywords:** prey naïveté, predator–prey, invasive mammalian predators, olfaction, feral cat, red fox

## Abstract

Invasive mammalian predators are linked to terrestrial vertebrate extinctions worldwide. Prey naïveté may explain the large impact invasive predators have on native prey; prey may fail to detect and react appropriately to the cues of novel predators, which results in high levels of depredation. In Australia, the feral cat (*Felis catus*) and the red fox (*Vulpes vulpes*) are implicated in more than 30 animal extinctions and the naïveté of native prey is often used to explain this high extinction rate. Reptiles are one group of animals that are heavily preyed upon by *F. catus* and *V. vulpes*. However, very few studies have examined whether reptiles are naive to their cues. In this study, we examine the ability of two native reptile species (*Morethia boulengeri* and *Christinus marmoratus*) to detect and distinguish between the chemical cues of two invasive predators (*V. vulpes* and *F. catus*) and three native predators (spotted-tailed quoll, *Dasyurus maculatus;* dingo, *Canis lupus dingo*; eastern brown snake, *Pseudonaja textilis*), as well as two non-predator controls (eastern grey kangaroo, *Macropus giganteus* and water). We conducted experiments to quantify the effects of predator scents on lizard foraging (the amount of food eaten) during 1 h trials within Y-maze arenas. We found both study species reduced the amount they consumed when exposed to predator scents—both native and invasive—indicating that these species are not naive to invasive predators. An evolved generalized predator-recognition system, rapid evolution or learned behaviour could each explain the lack of naïveté in some native Australian reptiles towards invasive predators.

## 1. Introduction

Predation is a strong selection pressure that drives the evolution of prey [1]. Prey species that have a shared evolutionary history with a predator tend to evolve ways to recognize their presence and respond through behaviours that minimize the risk of predation [2,3]. After detecting a predator, prey species can respond by moving to a secure location [4] and reducing their activity in the area [5]. Such shifts in behaviour are aimed at reducing the likelihood of encountering a predator and are thus collectively termed ‘anti-predator responses'.

Olfaction is a primary means by which prey animals detect predators [6]. Terrestrial predators often deliberately deposit strong smelling substances—including faeces, urine or secretions from specialized glands—throughout an animal's territory [7], allowing prey species to detect predators and trigger anti-predator responses [8]. However, in the absence of a shared evolutionary history between a prey and predator, a predator's scent may not be recognized by prey, and therefore, the anti-predator behaviour may not be employed [9]. Prey that lack the ability to read the cues of predators are said to be ‘naive’ [9]. Prey naïveté is highly problematic when a predator is introduced to a new region, as it can expose native prey to an enhanced risk of depredation [10,11]. This may explain why invasive predators have had such a devastating impact on native prey—for example, Doherty *et al*. [12] found that invasive mammalian predators are implicated in 58% of modern extinctions of birds, mammals and reptiles.

Australia is a model system for examining prey naïveté [13], due to the presence of a historic predator guild that included both marsupial and eutherian carnivores, and a contemporary predator guild dominated by two invasive, eutherian predators, the feral cat (*Felis catus*) and red fox (*Vulpes vulpes*). However, research on the naïveté of native prey to invasive predators has been dominated by studies of mammal prey (summarized in [13]). By contrast, the question of prey naïveté in reptiles has received very little attention. This is despite reptiles often comprising a large proportion of the diets of invasive mammalian predators—Doherty *et al*. [14] showed that feral cats in Australia consumed 157 species of reptile from nine families, and on average reptiles were found in a quarter of all sample units (feral cat stomachs or scats), with greater than 70% containing reptiles in the arid zone. Reptiles may be naive towards invasive predators and this may explain the high abundance of reptiles in their diets. There is some evidence to support this—Spencer [11] found that Murray River turtles (*Emydura macquarii*) failed to change their nesting behaviour to minimize the predation risk of offspring when exposed to olfactory cues of introduced red foxes, but did change their behaviour when exposed to olfactory cues of the native eastern quoll (*Dasyurus viverrinus*).

Here, we aim to determine if two species of native Australian reptile are naive to the olfactory cues of the feral cat and red fox. To do so, we examine whether individuals of each species respond behaviourally to the olfactory cues of cats, foxes and three native predators. Lizards were sourced from the Murray River catchment within the southwest slopes bioregion of New South Wales (NSW), southeastern Australia. Historically, this region had several native mammalian predators, including the spotted-tail quoll (*Dasyurus maculatus*) and the dingo (*Canis lupus dingo*), both of which are known to depredate upon lizards [15–17]. In the last 150 years, *V. vulpes* and *F. catus* have been introduced and are now both common throughout the region (*V. vulpes* has occurred within the study area since the early 1900s and *F. catus* since the mid-1800s [18]).

We exposed individuals from two lizard species—Boulenger's skink (*Morethia boulengeri*) and the southern marbled gecko (*Christinus marmoratus*)—to olfactory cues of predators to examine how lizards alter their foraging behaviour (proportion of food consumed) in the presence of these odours. The decision to focus on foraging is based on foraging theory, which predicts an individual will reduce or cease to forage when the reward is outweighed by the perceived predation risk [19,20]. Thus, if an individual perceives an area as risky, it will trigger an anti-predator response and cease or reduce time foraging. However, if a lizard (or any animal) is naive to the potential risk, the scent of a predator will not trigger an anti-predator response and, ceteris paribus, the lizard will forage similarly in the presence or absence of the scent. Therefore, the difference in foraging between areas with and without a predator scent is a measure of the risk that an animal associates with the scent. As lizards often avoid areas containing the scent of a predator with which they share an evolutionary history [4,21,22], it was predicted that lizards would consume less food in areas treated with the scents of native predators. By contrast, due to the lack of a shared evolutionary history with *V. vulpes* and *F. catus*, it was predicted that lizards would be naive to invasive predators, and therefore forage similarly in areas treated with and without the scents of invasive predators.

## 2. Material and methods

### 2.1. Study species

Two lizard species were chosen that vary in size, foraging behaviour, activity (diurnal and nocturnal) and habitat preferences. Both species are known to be preyed upon by invasive predators [14,23–26]. Boulenger's skink (*M. boulengeri*) is a small (SVL ∼ 50 mm), diurnal skink found within dry forests, box gum woodlands, river red gum forests and mallee woodland [27]. *Morethia boulengeri* often shelters beneath fallen timber, debris, leaf litter, surface rocks and exfoliating bark, and feeds upon small insects [27]. The southern marbled gecko (*C. marmoratus*) is a small (SVL ∼ 70 mm), nocturnal, arboreal gecko found within dry forests, box gum woodlands, river red gum forests and mallee woodlands. It is often located beneath exfoliating bark, in rock crevices and human dwellings, and feeds upon small insects [27].

### 2.2. Animal capture

Individual *M. boulengeri* and *C. marmoratus* were captured from the wild by hand. Adult animals were sexed. We retained males and non-gravid females with full tails for this study, whereas juveniles, gravid females and individuals that dropped their tails were immediately released. The point of capture was recorded by GPS and flagged using fluorescent tape. A total of 17 *M. boulengeri* (8 males, 9 females) were collected from a private property north of Rand (146.58° E, 35.59° S), Walla Swamp (Gum reserve) and another property south of Walla Walla (146.90° E, 35.77° S). A total of 27 individuals of *C. marmoratus* (11 males, 16 females) were collected from the wild at Charles Sturt University's Albury campus (146.99° E, 36.04° S) and Bells Travelling Stock Route (146.99° E, 36.039° S). At the completion of the study, all individuals were released at their point of capture. Details on animal husbandry are provided in electronic supplementary material, appendix S1.

### 2.3. Scent treatments

The study species were exposed to seven scent treatments—two were derived from invasive mammalian predators; two from native mammalian predators; one from a native reptilian predator; a procedural control; and a true control. The two invasive mammalian predators were the cat (*F. catus*) and the red fox (*V. vulpes*). Both species have been implicated in many Australian extinctions [12] and are widespread throughout the study region. The two native mammalian predators were the dingo (*C. l. dingo*) and spotted-tail quoll (*D. maculatus*). Both species occurred throughout the Murray catchment prior to European colonization (approx. 200 years ago) but are now regionally rare. The eastern brown snake (*Pseudonaja textilis*) was included as a native reptilian predator that is currently commonly found throughout the Murray catchment [27]. The procedural control was the scent of the eastern grey kangaroo (*Macropus giganteus*), a native marsupial herbivore that is common throughout the Murray catchment. This species was incorporated as a procedural control as their scent should not be associated with danger. Tap water was used as a true control. Faeces were used for species' scents, collected from deceased and living individuals (electronic supplementary material, appendix S2). A faeces solution for each species was made by mixing 30 g of scat with 250 ml of water. Samples from different individuals within the same species were mixed together to account for differences between the scents of males and females. The solution sat for 24 h before being strained through a cloth to remove solid particles and limit visual cues. The solution was poured into a spray bottle, which was set to misting for use in the trial arena.

### 2.4. Experimental design

To examine the response of lizards to the different scent treatments, a Y-shaped arena was used, hereafter referred to as a Y-maze. A Y-maze provides a ‘choice’ arena with a start arm, where the animal is positioned at the beginning of a trial and faced with the choice of remaining where it is or entering either of two longer arms. The majority of laboratory olfactory studies use Y-maze designs because the shape of the Y-maze reduces the chance that the scent will contaminate the entire study arena [28,29]. The design of the Y-maze for this study was adapted from [29]. Each arm of the Y-maze was 60 cm long and 20 cm wide, on a +45°/−45° angle from the start arm, which was 20 cm long and 20 cm wide. The width of the arms (20 cm) allowed individuals ample space to turn around without being hindered by the walls. The walls themselves were 20 cm high. Cling wrap was used to cover the Y-mazes to prevent animals from escaping during the trials. To account for lateralization bias, we randomized the arm the scent would be applied to using the random binary generator in Excel.

#### 2.4.1. Scent experiments

Each trial lasted 1 h. A tray of the species' preferred food (see electronic supplementary material, appendix S3) and a dish of water were placed at the ends of the two long arms of the Y-arena, with no food or water located in the start arm. In one arm of the arena, which was randomly selected across trails, 5 ml of a scent solution was applied with a spray bottle, while the other arm remained unscented. Animals were released into the start arm and therefore required to choose between the scented and unscented arms of the arena to obtain food or water. This follows an experimental approach used in previous studies [28–30]. *Morethia boulengeri* and *C. marmoratus* were offered three insects in each arm. The proportion of insects eaten within each arm was recorded at the end of the 1 h period.

We initially conducted these same experiments with a shelter included in each arm of the Y-maze (outlined in electronic supplementary material, appendix S4). Animals rarely left their shelters during the trials, and therefore did not consume much food in either arm of the Y-maze. Therefore, the shelters were removed from the Y-mazes to encourage foraging. Six trials were undertaken simultaneously in two temperature-controlled rooms, set to 23°C. Each of the species become active when temperatures exceed 17°C [31,32]. The scent treatment applied in each trial was randomly selected to ensure that trials occurring in the same room were not always accompanied by the same set of scents. This meant that some individuals underwent multiple trials for the same treatment, and not all individuals were exposed to all treatments (electronic supplementary material, table S1). Although the rooms were temperature controlled, there was variation throughout the study period—some particularly hot and cold days resulted in temperatures above or below the set temperature, resulting in some variability in average temperature. Therefore, we recorded ambient temperature during the trial in order to account for any variability during statistical analyses. At the completion of the 1 h trial period, the animals were returned to their home tanks. An hour was given between studies to allow time to clean the arenas between each trial to reduce the chances of contamination between scents. Each individual had at least a 24 h rest period between trials.

### 2.5. Data analysis

All analyses were conducted within R v. 3.4.0. Generalized linear mixed models (GLMMs) were used to compare the amount of food eaten in the scented arm compared to the amount eaten in the unscented (i.e. the control arm). GLMMs were required because they allow for non-normally distributed response variables and random effects that account for dependency among measurements (i.e. repeated measures of the same individual; [33]). In this instance, the response variable was proportion data (the proportion of food eaten versus the proportion of food remaining in each arm at the end of the trial, modelled as the number of successes and failures among a fixed number of Bernoulli trials), bound at zero and one, and was therefore assumed to follow a binomial distribution [34]. Random effects were required because the same individuals were included in multiple trials, and because two observations were taken within each trial (i.e. one of the scented arm and one of the unscented arm). Therefore, an ID number of each individual and the experiment trial number were included as random effects to account for non-independence in the data [33]. GLMMs were fitted using the R package ‘lme4’ v. 1.1–13 [35]. We initially included the trial number for each individual as a covariate in order to account for possible carryover effects. However, this variable was not significant and so was not included in the final analysis. Tests for over-dispersion were undertaken to assess whether there was additional variance in the data than assumed by the binomial distribution. If models were over-dispersed, an additional observation-level random effect was included to correct for over-dispersion [36]. Model fit was assessed using marginal *R*^2^ values [37].

A set of four candidate GLMMs was constructed for each study species. All models included the true control and the procedural control, the average temperature during the trial and the sex of the study animal. Models differed only with regard to how predator scents are grouped in order to reveal the level at which study species distinguish between predators. The first model grouped all predator scents into a single category. We refer to this as the ‘general predator model’ as it would be supported if lizards responded in a general (i.e. uniform) way to all predators, regardless of their origin (i.e. native versus invasive) or taxonomy (i.e. mammal versus reptile). The second model collapsed the three native predators (*C. dingo*, *D. maculatus* and *P. textilis*) into a single category (native predators) and collapsed *V. vulpes* and *F. catus* into a separate category (invasive predators). We refer to this as the ‘predator origin model’, as this model would be supported when the study species respond differently to predators depending on their geographic origin (i.e. distinguishing between native and invasive predators). The third model grouped the predator species by their phylogenetic relatedness rather than their geographic origin, thus grouping *C. dingo*, *D. maculatus*, *V. vulpes* and *F. catus* into a single ‘mammalian predator’ category, leaving *P. textilis* in a separate category. We refer to this as the ‘predator phylogeny model’, as it would be supported if the study species distinguish between the mammalian and reptilian predators (regardless of their origin). The fourth model included a categorical variable with six levels: *C. l. dingo* scent, *D. maculatus* scent, *V. vulpes* scent, *F. catus* scent, *P. textilis* scent, herbivore scent (procedural control) and misted water (true control). This model is called the ‘predator species model’ as it would be supported when the study species respond differently to each species' scent.

Akaike's information criterion adjusted for small sample bias (AICc) was used to compare the level of support for models within the candidate set [38]. Models that receive the lowest AICc values have the most support, given the data. Akaike weights (*w_i_*) were calculated to provide an estimate of the probability that any given model was the best within the candidate set [38]. AICc differences (Δ*_i_*)—the difference between the AICc with the lowest value and all other AICc values—were calculated to examine the level of support for models other than the most supported model [38]. Models with Δ*_i_* values <2 are considered to have substantial support [38]. Therefore, parameter estimates (coefficients and standard errors) were inspected for all models with Δ*_i_* < 2. Depending on the direction and size of the parameter effects, support for the general predator and predator phylogeny models would imply that the study species are not naive to invasive predators (i.e. responding in a similar way regardless of predator origin), whereas support for the predator origin and predator species models would imply that study species are responding differently to invasive and native predators or in a species-specific way, and thus could equate to naïveté.

## 3. Results

The trials comprised 17 *M. boulengeri* exposed to 54 trials and 27 *C. marmoratus* exposed to 81 trials. An average of 0.62 insects was eaten by *M. boulengeri* in the control arms (range = 0–3), and 0.13 insects eaten from predator-scented arms (range = 0–2). At least one insect was removed in 46% of the control arms, while at least one insect was removed in 10% of predator-scented arms. An average of 1.36 insects was eaten by *C. marmoratus* in the control arms (range = 0–3), compared to 0.59 insects in the predator-scented arms (range = 0–3). At least one insect was removed in 75% of the control arms, while at least one insect was removed in 40% of predator-scented arms.

The procedural control did not differ from the true control in any model for any species (table 2), indicating that it was not the application of an animal scent alone that elicited the responses.

**Figure 1.**
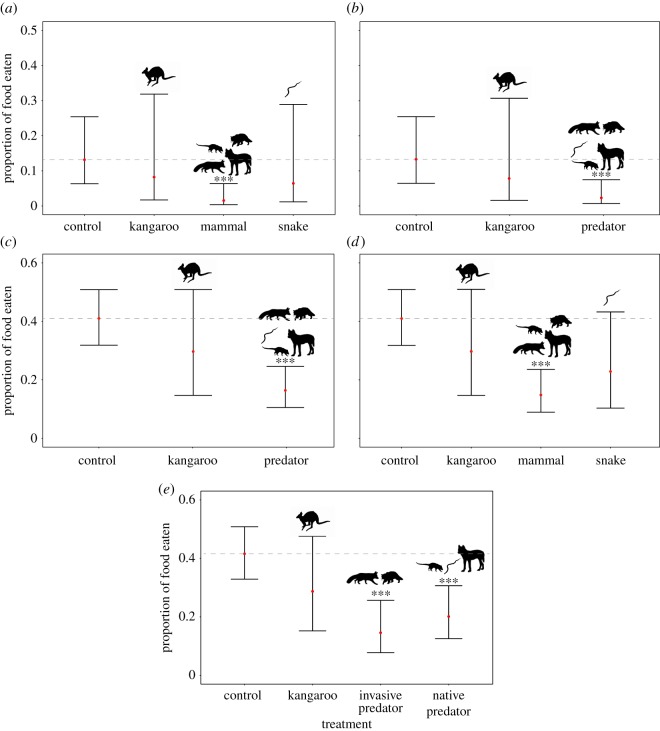
Responses of *M. boulengeri* (*a*,*b*) and *C. marmoratus* (*c*–*e*) to olfactory cues of native and invasive predators, displaying models with ΔAIC < 2 for both species. The response variable was the proportion of food consumed over standard trial time (1 h). The dotted line represents predicted average of the control, error bars depict 95% confidence intervals, red dots represent the point estimates and three asterisks depict that the treatment differs significantly from the control. *<0.05, **<0.01, ***<0.001).

Model selection for *M. boulengeri* showed that the general predator model and the predator phylogeny models had substantial support (table 1), suggesting this species responded to predators in a uniform way, but potentially distinguished between mammalian predators and *P. textilis*. These models explained a moderate proportion of the variance in the data—between 15 and 20% (table 1). Parameter estimates showed that *M. boulengeri* individuals significantly reduced the amount of food consumed when exposed to the scent of mammalian predators (regardless of origin) compared to the control (table 2 and figure 1*a,b*). Sex and temperature had no effect on the amount of food consumed (table 2).

**Table 1.** Model selection of generalized linear mixed models representing hypotheses of the level at which native lizard species distinguish between the scents of predator species. Included are the number of parameters (*K*), log-likelihood of the model (logLik), AICc values, AICc differences (Δ*_i_*), Akaike weights (*w_i_*) and model fit (*R*^2^m). Models for which AICc differences are less than 2.0 are shown in italics.

species	model	*K*	logLik	AICc	Δ*_i_*	*w_i_*	*R*^2^m
*M. boulengeri*	*general predator model*	*7*	*−60.68*	*136.71*	*0.00*	0.46	*0.19*
	*predator phylogeny model*	*8*	*−59.80*	*137.33*	*0.62*	0.34	*0.15*
	predator origin model	8	−60.68	139.10	2.39	0.14	0.15
	predator species model	11	−57.81	140.93	4.21	0.06	0.20
*C. marmoratus*	*general predator model*	*7*	*−163.93*	*342.74*	*0.00*	*0.49*	*0.13*
	*predator phylogeny model*	*8*	*−163.45*	*344.04*	*1.30*	*0.26*	*0.14*
	*predator origin model*	*8*	*−163.55*	*344.24*	*1.50*	*0.23*	*0.13*
	predator species model	11	−162.63	349.39	6.65	0.02	0.15

**Table 2.** Parameter estimates from generalized linear mixed models with support for each species. Scent treatments that differed from the control (i.e. 95% confidence interval not overlapping zero and *p*-value < 0.05) are shown in italics. Models are in rank order from model selection.

species	model	Parameters	coefficient	s.e.	*Z*	*p-*value
*M. boulengeri*	general predator model	intercept	−1.82	0.52	−3.48	0.00
		*predator*	*−1.87*	*0.55*	*−3.39*	*0.00*
		kangaroo	−0.59	0.84	−0.70	0.48
		temperature	−0.18	0.23	−0.79	0.43
		sex (M)	−0.13	0.73	−0.19	0.85
	predator phylogeny model	intercept	−1.82	0.53	−3.44	0.00
		kangaroo	−0.53	0.85	−0.62	0.53
		*mammal*	*−2.27*	*0.68*	*−3.35*	*0.00*
		snake	−0.79	0.87	−0.91	0.36
		temperature	−0.15	0.23	−0.67	0.51
		sex (M)	−0.17	0.74	−0.23	0.82
*C. marmoratus*	general predator model	intercept	−0.11	0.24	−0.43	0.67
		*predator*	−*1.27*	*0.27*	−*4.71*	*0.00*
		kangaroo	−0.50	0.47	−1.07	0.29
		*temperature*	−*0.32*	*0.16*	−*2.04*	*0.04*
		sex (M)	−0.68	0.36	−1.86	0.06
	predator phylogeny model	intercept	−0.11	0.25	−0.43	0.66
		kangaroo	−0.50	0.47	−1.06	0.29
		*mammal*	*−1.38*	*0.30*	*−4.64*	*0.00*
		snake	−0.85	0.49	−1.75	0.08
		*temperature*	*−0.33*	*0.16*	*−2.08*	*0.04*
		sex (M)	−0.67	0.37	−1.84	0.07
	predator origin model	intercept	−0.10	0.24	−0.41	0.68
		kangaroo	−0.50	0.47	−1.07	0.28
		*invasive*	*−1.51*	*0.40*	*−3.80*	*0.00*
		*native*	*−1.11*	*0.32*	*−3.45*	*0.00*
		*temperature*	*−0.32*	*0.16*	*−2.04*	*0.04*
		sex (M)	−0.69	0.36	−1.88	0.06

Model selection for *C. marmoratus* showed that three models had substantial support (table 1)—the general predator model, the predator phylogeny model and the predator origin model. Parameter estimates showed that *C. marmoratus* significantly reduced the amount of food consumed when exposed to the scent of mammalian predators compared to the control (table 2 and figure 1). Parameter estimates from the predator origin model showed that *C. marmoratus* reduced its foraging in the presence of both invasive and native predators (table 2), but the effect was stronger for the former than the latter. The amount of food consumed in arms treated with *P. textilis* scent did not differ significantly from the amount consumed in the control arm (table 2). Temperature had a negative effect on the proportion of food consumed—the warmer it was, the less food was consumed (table 2).

## 4. Discussion

The ability of prey to accurately recognize olfactory cues and deploy anti-predator response is vital for avoiding predation, yet prey that have not co-evolved with a particular predator species may be naive to olfactory cues [9]. A key finding of our study is that both prey species responded to the olfactory cues of invasive predators in a way that is likely to diminish the risk of predation. Thus, we reject our hypothesis that the two study species would be naive to the olfactory cues of invasive predators due to their relatively brief history of co-occurrence. Given that *M. boulengeri* and *C. marmoratus* lack naïveté, what mechanisms might have led to the development of these anti-predator responses, particularly given the relatively short history of co-occurrence with the two invasive predators?

First, despite our study species having a short history of co-occurrence with *F. catus* and *V. vulpes* (approximately 150 years)*,* each has a relatively long history of co-evolution with other mammalian predators, including *C. l. dingo* and *D. maculatus*. Blumstein's [39] multi-predator hypothesis suggests that animals co-occurring with multiple predators do not evolve species-specific systems for recognizing predators, but instead develop generalized recognition systems for a range of morphologically or behaviourally similar predators [39]. Thus, the ability of *M. boulengeri* and *C. marmoratus* to detect and respond to the olfactory cues of *F. catus* and *V. vulpes* could be due to their shared history with *C. l. dingo* and *D. maculatus*. Despite being clearly distinct in their phylogeny and place of origin, all of our mammalian predator species are medium-sized carnivores, and it is possible their olfactory cues are similar enough that native prey generally recognize them as predators [40,41]. Studies have shown 2-phenylethylamine to be common in the urine of many carnivore species, and rodents exposed to 2-phenylethylamine display hard-wired avoidance behaviours [41]. Do similar compounds exist in the faeces of our study predators that elicit the generalized behavioural responses we observed? While further research is required to answer this question, our findings support the notion of a generalized predator response as the models that combined all mammalian predators into one group were often among the most supported. Similar results were observed by Carthey and Banks [42], who found that bush rats (*Rattus fuscipes*)—a native Australian rodent—were more vigilant and foraged less when exposed to the olfactory cues of *F. catus* and *V. vulpes*, despite a lack of long-term shared evolutionary history [42].

A second possibility is that 150 years of shared evolutionary history with invasive mammalian predators has been sufficient for these two lizards to evolve behavioural adaptations that reduce predation from these invasive predators [1]. Predation is a significant selective pressure and, as such, mortality through predation is a strong driver of evolution [43]. Many Australian lizard species have short generational times allowing for the potential for natural selection to occur over 150 years. *Christinus marmoratus* reach sexual maturity at 2–3 years of age. If reproduction occurred at least once in their lifetime after they reached sexual maturation, a minimum of 50 generations could have occurred over 150 years. *Morethia boulengeri* reaches sexual maturity at approximately 9 months, meaning a minimum of 150 generations could have occurred (allowing for seasonailty of breeding) in the presence of *F. catus* and *V. vulpes* (Henle [32]). Nunes *et al*. [1] examined five populations of water frog, *Pelophylax perezi*, from different historical exposures (30 years, 20 years or no coexistence) to an invasive predator, the red swamp crayfish (*Procambarus clarkia*). *Pelophylax perezi* populations that had co-occurred with *P. clarkia* for 20–30 years—equivalent to just 10–15 *P. perezi* generations—already demonstrated both morphological and behavioural adaptations that reduced predation risk. This suggests invasive predators may drive rapid evolutionary change through natural selection [1].

A third possibility is that the responses are due to learned behaviours [44]. As our study animals were collected from the wild in areas where *F. catus* and *V. vulpes* are common, it is possible that these animals had learned to associate the olfactory cues of both species with a risk of predation through prior experience. Indeed, a single negative experience can create long-term aversion [44], and this could also be true for prey that have had a negative experience with a predator. For example, Ortegar *et al*. [45] showed that wall lizards (*Podarcis pityusensis*) could recognize the scent of the invasive horseshoe whip snake (*Hemorrhois hippocrepis*) and deploy anti-predator behaviours in response, despite having a very short period of co-occurrence [45]. They concluded the lizards had learned to associate the scent with the risk of predation.

An alternative explanation to our observations is that the study species did not respond in a fearful way to the predator scents at all, but rather that they exhibited disgust [46]. Predator faeces are known to host pathogens that could infect lizards, and lizards therefore may choose to avoid areas with faeces to avoid infection rather than predation [47]. Such avoidance behaviour can lead to a landscape of ‘disgust’ that overlays landscapes of fear to shape prey movement patterns [46]. However, even if disgust behaviour is primarily aimed at reducing infection risk, it may well help to diminish predation risk as well [47]. If the responses we observed are due to disgust, it is nonetheless interesting that the study species were able to identify the faeces of invasive species as an infection risk, given their brief history of evolutionary co-occurrence. The mechanisms that could lead prey to recognize the faeces of invasive predators as an infection risk are largely the same as those outlined above (i.e. a generalized response, natural selection or learned behaviour). Identifying the underlying motivations for behaviours remains a significant challenge in ecology, particularly when very different motivations could lead to the same patterns of behaviour.

Presuming that the motivation of the responses is fear and not disgust, we conclude that the most likely explanations for the lack of naïveté in our study species are either: (i) a generalized predator-recognition system that individuals are applying to the new predators, (ii) rapid evolution of behavioural adaptations caused by the strong natural selection of predation, or (iii) a learned behaviour derived from past encounters with the predators. Future research could help distinguish between these hypotheses by examining whether responses of individuals differ according to the amount of time that their populations have been exposed to invasive predators, or, where possible, by comparing responses of individuals from populations that have had no history of co-occurrence with these predators (e.g. populations on islands that have not been colonized by foxes and cats) compared to those that have approximately 150 years of co-occurrence (e.g. mainland populations). A greater understanding of the processes that diminish naïveté of prey to invasive predators could help to predict or ameliorate the impacts of invasive species.

## Supplementary Material

Appendix S1; Appendix S2; Appendix S3; Appendix S4; Table S1

## Data accessibility

Data are provided as a supplement (electronic supplementary material, table S1).
