# Supplementary material for: Native reptiles alter their foraging in the presence of the olfactory cues of invasive mammalian predators
Source: R Soc Open Sci. 2018 Oct 31;5(10):180136. doi: 10.1098/rsos.180136 (PMC6227964; doi:10.1098/rsos.180136)
Supplement: Appendix S1; Appendix S2; Appendix S3; Appendix S4; Table S1 [file rsos180136supp1.docx]

**Appendix S1. Animal husbandry**

*M. boulengeri* individuals were maintained in individual open-topped glass tanks (45 x 35 x 28.5 cm). *M. boulengeri* were fed four to five small (~1 cm long) crickets three times a week and three small (~1 cm long) mealworms (*Tenebrio molitor*) twice a week. As *C. marmoratus* are arboreal and could escape the open-topped tanks, they were housed in plastic containers (5L) with air holes through both ends of the lid. *C. marmoratus* were fed one small (~1 cm long) cricket three times weekly, and two small (~1 cm long) mealworms three times a week. Sand substrate one cm deep with leaf litter and fallen bark was placed in the housing tanks or plastic containers. Artificial refuges made of black plastic food containers, placed upside down with a single entry point, were provided as shelter. Water was provided in a small dish and replenished daily. A temperature gradient of (20 – 35 °C) was achieved by a heating cord (9 m = 80 W) attached to the front exterior surface of each tank. UVB lights were provided by 1.2 m fluorescent tubes (36 W) mounted above the tanks. Heat cords and lighting ware switched on between 0800 and 1800 hrs to achieve an even 12 h light/darkness cycle to imitate day-and night exposure.

**Appendix S2. Predator scent collection**

*F. catus* scats were collected from deceased animals captured across the south-west slopes (SWS) of NSW (1 female and 3 individuals of unknown sex). Fresh scats were also collected from two male domestic *F. catus* from within the study region. *V. vulpes* faeces were also collected from deceased animals captured across the SWS of NSW (2 males, 1 female) and from living animals located within the study region (unknown sex, unknown age of scats). Fresh dingo/wild dog scats were collected from male and female individuals in captivity (2 males, 2 females) from Cooberrie Park Wildlife Sanctuary, Woodbury, Queensland, and one living wild male from Rand, NSW (scats were found in the field, of unknown age). Fresh *D. maculatus* scats (2 males, 2 females) were collected from Featherdale Wildlife Park, Doonside, NSW. Fresh eastern brown snake scat was collected from a single captive animal (sex unknown) in Queensland. Eastern grey kangaroo scats were collected from from Rand (unknown sex, unknown age of scats), NSW and Gum reserve, NSW. Scats were stored in zip lock bags on ice in a polystyrene foam box and transported to freezers set to -20°C.

**Appendix S3. Food preference trials**

Pilot studies on food preference were undertaken to increase the chance that animals would forage during the scent experiment by providing each species with its preferred food. Food preference trials consisted of two, twenty minute trials per individual, in which animals were released into the start arm of the Y-maze with two different food choices in each arm. The foods offered to each species were based on knowledge of their diets derived from field guides [1] and experts (J. Riley, personal communication, November 2016). The amount and type of food eaten was monitored to determine which foods were being favoured. *M. boulengeri* and *C. marmoratus* were offered crickets and mealworms, *M. boulengeri* were exposed to 34 trials which indicated that they preferred crickets in 70% of trials. *C. marmoratus* were exposed to 26 trials, which indicated they preferred meal worms in 65% of trials. Therefore, crickets and meal worms were used during Y-maze trials for *M. boulengeri* and *C. marmoratus*, respectively.

**References**

[1] Michael, D. & Lindenmayer, D. 2010 *Reptiles of the NSW Murray catchment: a guide to their identification, ecology and conservation*, CSIRO PUBLISHING.

**Appendix S4. Trials including a refuge**

Prior to the final experiments reported in the paper, we conducted some initial experiments on the two study species, as well as a further study species (*Egernia striolata*), that included refuges in either side of the Y-maze. This experiment included 17 *M. boulengeri* exposed to 54 trials; 27 *C. marmoratus* exposed to 81 trials, and 24 *E. striolata* exposed to 72 trials. *E. striolata. Egernia striolata* individuals (7 males, 17 females) were collected from the wild near Albury, NSW (146.90°E, 36.06°S). These individuals were maintained in captivity for 2 years prior to this study at Macquarie University. These animals were unavailable during the second experiment. This experiment followed the same procedures as the experiment reported in the paper, except that a shelter was placed in both arms of the Y maze (no shelter was placed in the start arm). *M. boulengeri* and *C. marmoratus* were offered three insects in each arm, while *E. striolata* was offered one teaspoon of baby food in each arm, spread thinly and evenly (~1 mm deep) across a shallow dish. The proportion of insects eaten or the proportion of the baby food removed (estimated visually as the proportion of the dish in which food had been removed) within each arm was recorded at the end of the one-hour period. At the completion of the one-hour trial, the animals were returned to their home tanks. Results from these trials indicated that individuals rarely moved outside of the shelters, leading to few relationships between foraging and scent treatments. Therefore, it was decided to modify the experimental design to increase the likelihood of animals engaging in foraging behaviour by removing the option of animals seeking shelter for the duration of the trial.

**Table S1.** Raw data used in the analysis presented in the manuscript. FE = food eaten during the trial, FR = food removed during the trial. Ave temp = the average temperature (°C) during the trial.

| **Species** | **ID** | **Sex** | **Trial** | **Scent** | **Treat** | **FE** | **FR** | **Ave temp** |
| --- | --- | --- | --- | --- | --- | --- | --- | --- |
| *Christinus marmortus* | 1 | f | 3 | Snake | C | 0 | 3 | 21.25 |
| *Christinus marmortus* | 1 | f | 3 | Snake | T | 0 | 3 | 21.25 |
| *Christinus marmortus* | 1 | f | 4 | Kangaroo | C | 0 | 3 | 21 |
| *Christinus marmortus* | 1 | f | 4 | Kangaroo | T | 0 | 3 | 21 |
| *Christinus marmortus* | 1 | f | 5 | Cat | C | 0 | 3 | 21.25 |
| *Christinus marmortus* | 1 | f | 5 | Cat | T | 0 | 3 | 21.25 |
| *Christinus marmortus* | 2 | f | 8 | Dingo | C | 3 | 0 | 21.25 |
| *Christinus marmortus* | 2 | f | 8 | Dingo | T | 0 | 3 | 21.25 |
| *Christinus marmortus* | 2 | f | 9 | Cat | C | 3 | 0 | 21 |
| *Christinus marmortus* | 2 | f | 9 | Cat | T | 1 | 2 | 21 |
| *Christinus marmortus* | 2 | f | 10 | Quoll | C | 3 | 0 | 21.25 |
| *Christinus marmortus* | 2 | f | 10 | Quoll | T | 0 | 3 | 21.25 |
| *Christinus marmortus* | 3 | m | 14 | Fox | C | 0 | 3 | 21.25 |
| *Christinus marmortus* | 3 | m | 14 | Fox | T | 0 | 3 | 21.25 |
| *Christinus marmortus* | 4 | m | 18 | Quoll | C | 1 | 2 | 21 |
| *Christinus marmortus* | 4 | m | 18 | Quoll | T | 0 | 3 | 21 |
| *Christinus marmortus* | 4 | m | 19 | Snake | C | 2 | 1 | 21.25 |
| *Christinus marmortus* | 4 | m | 19 | Snake | T | 1 | 2 | 21.25 |
| *Christinus marmortus* | 5 | m | 21 | Snake | C | 2 | 1 | 21 |
| *Christinus marmortus* | 5 | m | 21 | Snake | T | 1 | 2 | 21 |
| *Christinus marmortus* | 5 | m | 22 | Kangaroo | C | 1 | 2 | 21 |
| *Christinus marmortus* | 5 | m | 22 | Kangaroo | T | 2 | 1 | 21 |
| *Christinus marmortus* | 5 | m | 23 | Fox | C | 1 | 2 | 21.25 |
| *Christinus marmortus* | 5 | m | 23 | Fox | T | 0 | 3 | 21.25 |
| *Christinus marmortus* | 6 | f | 26 | Dingo | C | 3 | 0 | 21 |
| *Christinus marmortus* | 6 | f | 26 | Dingo | T | 3 | 0 | 21 |
| *Christinus marmortus* | 6 | f | 27 | Cat | C | 3 | 0 | 21 |
| *Christinus marmortus* | 6 | f | 27 | Cat | T | 2 | 1 | 21 |
| *Christinus marmortus* | 6 | f | 28 | Kangaroo | C | 1 | 2 | 21.25 |
| *Christinus marmortus* | 6 | f | 28 | Kangaroo | T | 0 | 3 | 21.25 |
| *Christinus marmortus* | 7 | f | 32 | Fox | C | 3 | 0 | 21 |
| *Christinus marmortus* | 7 | f | 32 | Fox | T | 1 | 2 | 21 |
| *Christinus marmortus* | 8 | f | 35 | Quoll | C | 2 | 1 | 21 |
| *Christinus marmortus* | 8 | f | 35 | Quoll | T | 0 | 3 | 21 |
| *Christinus marmortus* | 8 | f | 36 | Snake | C | 1 | 2 | 21 |
| *Christinus marmortus* | 8 | f | 36 | Snake | T | 0 | 3 | 21 |
| *Christinus marmortus* | 9 | m | 39 | Quoll | C | 1 | 2 | 21 |
| *Christinus marmortus* | 9 | m | 39 | Quoll | T | 2 | 1 | 21 |
| *Christinus marmortus* | 9 | m | 40 | Kangaroo | C | 0 | 3 | 21 |
| *Christinus marmortus* | 9 | m | 40 | Kangaroo | T | 1 | 2 | 21 |
| *Christinus marmortus* | 9 | m | 41 | Fox | C | 1 | 2 | 21 |
| *Christinus marmortus* | 9 | m | 41 | Fox | T | 0 | 3 | 21 |
| *Christinus marmortus* | 10 | m | 45 | Snake | C | 1 | 2 | 21 |
| *Christinus marmortus* | 10 | m | 45 | Snake | T | 0 | 3 | 21 |
| *Christinus marmortus* | 10 | m | 46 | Cat | C | 1 | 2 | 21 |
| *Christinus marmortus* | 10 | m | 46 | Cat | T | 0 | 3 | 21 |
| *Christinus marmortus* | 10 | m | 47 | Kangaroo | C | 1 | 2 | 21 |
| *Christinus marmortus* | 10 | m | 47 | Kangaroo | T | 0 | 3 | 21 |
| *Christinus marmortus* | 11 | f | 51 | Dingo | C | 0 | 3 | 21 |
| *Christinus marmortus* | 11 | f | 51 | Dingo | T | 1 | 2 | 21 |
| *Christinus marmortus* | 12 | f | 55 | Fox | C | 2 | 1 | 21 |
| *Christinus marmortus* | 12 | f | 55 | Fox | T | 0 | 3 | 21 |
| *Christinus marmortus* | 12 | f | 56 | Quoll | C | 2 | 1 | 21 |
| *Christinus marmortus* | 12 | f | 56 | Quoll | T | 0 | 3 | 21 |
| *Christinus marmortus* | 12 | f | 57 | Snake | C | 2 | 1 | 21 |
| *Christinus marmortus* | 12 | f | 57 | Snake | T | 2 | 1 | 21 |
| *Christinus marmortus* | 13 | f | 60 | Cat | C | 3 | 0 | 21 |
| *Christinus marmortus* | 13 | f | 60 | Cat | T | 0 | 3 | 21 |
| *Christinus marmortus* | 13 | f | 61 | Fox | C | 0 | 3 | 21 |
| *Christinus marmortus* | 13 | f | 61 | Fox | T | 1 | 2 | 21 |
| *Christinus marmortus* | 14 | f | 65 | Dingo | C | 1 | 2 | 21 |
| *Christinus marmortus* | 14 | f | 65 | Dingo | T | 0 | 3 | 21 |
| *Christinus marmortus* | 14 | f | 66 | Kangaroo | C | 0 | 3 | 21 |
| *Christinus marmortus* | 14 | f | 66 | Snake | C | 3 | 0 | 20.25 |
| *Christinus marmortus* | 14 | f | 67 | Kangaroo | T | 0 | 3 | 21 |
| *Christinus marmortus* | 14 | f | 67 | Snake | T | 0 | 3 | 20.25 |
| *Christinus marmortus* | 15 | f | 70 | Fox | C | 1 | 2 | 21.25 |
| *Christinus marmortus* | 15 | f | 70 | Fox | T | 0 | 3 | 21.25 |
| *Christinus marmortus* | 15 | f | 71 | Quoll | C | 3 | 0 | 21.25 |
| *Christinus marmortus* | 15 | f | 71 | Quoll | T | 0 | 3 | 21.25 |
| *Christinus marmortus* | 15 | f | 72 | Cat | C | 0 | 3 | 20.25 |
| *Christinus marmortus* | 15 | f | 72 | Cat | T | 0 | 3 | 20.25 |
| *Christinus marmortus* | 16 | f | 76 | Snake | C | 0 | 3 | 21.25 |
| *Christinus marmortus* | 16 | f | 76 | Snake | T | 0 | 3 | 21.25 |
| *Christinus marmortus* | 16 | f | 77 | Kangaroo | C | 3 | 0 | 21.25 |
| *Christinus marmortus* | 16 | f | 77 | Kangaroo | T | 1 | 2 | 21.25 |
| *Christinus marmortus* | 16 | f | 78 | Fox | C | 0 | 3 | 21 |
| *Christinus marmortus* | 16 | f | 78 | Fox | T | 0 | 3 | 21 |
| *Christinus marmortus* | 17 | m | 81 | Dingo | C | 0 | 3 | 21.25 |
| *Christinus marmortus* | 17 | m | 81 | Dingo | T | 1 | 2 | 21.25 |
| *Christinus marmortus* | 17 | m | 82 | Cat | C | 1 | 2 | 21.25 |
| *Christinus marmortus* | 17 | m | 82 | Cat | T | 1 | 2 | 21.25 |
| *Christinus marmortus* | 17 | m | 83 | Kangaroo | C | 0 | 3 | 21 |
| *Christinus marmortus* | 17 | m | 83 | Kangaroo | T | 0 | 3 | 21 |
| *Christinus marmortus* | 18 | m | 87 | Quoll | C | 0 | 3 | 21 |
| *Christinus marmortus* | 18 | m | 87 | Quoll | T | 1 | 2 | 21 |
| *Christinus marmortus* | 19 | m | 90 | Fox | C | 1 | 2 | 21.25 |
| *Christinus marmortus* | 19 | m | 90 | Fox | T | 1 | 2 | 21.25 |
| *Christinus marmortus* | 19 | m | 91 | Quoll | C | 1 | 2 | 21 |
| *Christinus marmortus* | 19 | m | 91 | Quoll | T | 0 | 3 | 21 |
| *Christinus marmortus* | 19 | m | 92 | Dingo | C | 1 | 2 | 21 |
| *Christinus marmortus* | 19 | m | 92 | Dingo | T | 1 | 2 | 21 |
| *Christinus marmortus* | 20 | f | 96 | Snake | C | 1 | 2 | 21.25 |
| *Christinus marmortus* | 20 | f | 96 | Snake | T | 2 | 1 | 21.25 |
| *Christinus marmortus* | 20 | f | 97 | Kangaroo | C | 0 | 3 | 21 |
| *Christinus marmortus* | 20 | f | 97 | Kangaroo | T | 0 | 3 | 21 |
| *Christinus marmortus* | 20 | f | 98 | Fox | C | 2 | 1 | 20.5 |
| *Christinus marmortus* | 20 | f | 98 | Fox | T | 0 | 3 | 20.5 |
| *Christinus marmortus* | 21 | f | 101 | Dingo | C | 0 | 3 | 21.25 |
| *Christinus marmortus* | 21 | f | 101 | Dingo | T | 2 | 1 | 21.25 |
| *Christinus marmortus* | 21 | f | 102 | Cat | C | 1 | 2 | 21 |
| *Christinus marmortus* | 21 | f | 102 | Cat | T | 2 | 1 | 21 |
| *Christinus marmortus* | 21 | f | 103 | Kangaroo | C | 1 | 2 | 20.5 |
| *Christinus marmortus* | 21 | f | 103 | Kangaroo | T | 3 | 0 | 20.5 |
| *Christinus marmortus* | 22 | m | 107 | Snake | C | 0 | 3 | 21.25 |
| *Christinus marmortus* | 22 | m | 107 | Snake | T | 0 | 3 | 21.25 |
| *Christinus marmortus* | 22 | m | 108 | Quoll | C | 1 | 2 | 20.5 |
| *Christinus marmortus* | 22 | m | 108 | Quoll | T | 0 | 3 | 20.5 |
| *Christinus marmortus* | 23 | m | 111 | Cat | C | 2 | 1 | 21.25 |
| *Christinus marmortus* | 23 | m | 111 | Cat | T | 0 | 3 | 21.25 |
| *Christinus marmortus* | 23 | m | 112 | Quoll | C | 1 | 2 | 20.25 |
| *Christinus marmortus* | 23 | m | 112 | Quoll | T | 0 | 3 | 20.25 |
| *Christinus marmortus* | 23 | m | 113 | Dingo | C | 2 | 1 | 20.5 |
| *Christinus marmortus* | 23 | m | 113 | Dingo | T | 0 | 3 | 20.5 |
| *Christinus marmortus* | 24 | m | 116 | Kangaroo | C | 1 | 2 | 20.25 |
| *Christinus marmortus* | 24 | m | 116 | Kangaroo | T | 1 | 2 | 20.25 |
| *Christinus marmortus* | 24 | m | 117 | Fox | C | 2 | 1 | 19.75 |
| *Christinus marmortus* | 24 | m | 117 | Fox | T | 0 | 3 | 19.75 |
| *Christinus marmortus* | 25 | f | 121 | Fox | C | 0 | 3 | 21 |
| *Christinus marmortus* | 25 | f | 121 | Fox | T | 2 | 1 | 21 |
| *Christinus marmortus* | 25 | f | 122 | Cat | C | 2 | 1 | 20.25 |
| *Christinus marmortus* | 25 | f | 122 | Cat | T | 0 | 3 | 20.25 |
| *Christinus marmortus* | 25 | f | 123 | Kangaroo | C | 3 | 0 | 19.75 |
| *Christinus marmortus* | 25 | f | 123 | Kangaroo | T | 3 | 0 | 19.75 |
| *Christinus marmortus* | 26 | f | 127 | Snake | C | 2 | 1 | 21 |
| *Christinus marmortus* | 26 | f | 127 | Snake | T | 1 | 2 | 21 |
| *Christinus marmortus* | 26 | f | 128 | Snake | C | 3 | 0 | 20.25 |
| *Christinus marmortus* | 26 | f | 128 | Snake | T | 2 | 1 | 20.25 |
| *Christinus marmortus* | 26 | f | 129 | Quoll | C | 1 | 2 | 19.75 |
| *Christinus marmortus* | 26 | f | 129 | Quoll | T | 2 | 1 | 19.75 |
| *Christinus marmortus* | 27 | f | 132 | Dingo | C | 0 | 3 | 21 |
| *Christinus marmortus* | 27 | f | 132 | Dingo | T | 0 | 3 | 21 |
| *Christinus marmortus* | 27 | f | 133 | Cat | C | 2 | 1 | 20.25 |
| *Christinus marmortus* | 27 | f | 133 | Cat | T | 1 | 2 | 20.25 |
| *Christinus marmortus* | 27 | f | 134 | Dingo | C | 2 | 1 | 19.75 |
| *Christinus marmortus* | 27 | f | 134 | Dingo | T | 0 | 3 | 19.75 |
| *Morethia boulengeri* | 1 | m | 3 | Dingo | C | 1 | 2 | 23 |
| *Morethia boulengeri* | 1 | m | 3 | Dingo | T | 0 | 3 | 23 |
| *Morethia boulengeri* | 1 | m | 4 | Dingo | C | 1 | 2 | 23 |
| *Morethia boulengeri* | 1 | m | 4 | Dingo | T | 0 | 3 | 23 |
| *Morethia boulengeri* | 2 | m | 8 | Quoll | C | 0 | 3 | 23 |
| *Morethia boulengeri* | 2 | m | 8 | Quoll | T | 0 | 3 | 23 |
| *Morethia boulengeri* | 2 | m | 9 | Quoll | C | 0 | 3 | 23 |
| *Morethia boulengeri* | 2 | m | 9 | Quoll | T | 0 | 3 | 23 |
| *Morethia boulengeri* | 2 | m | 10 | Fox | C | 1 | 2 | 23.25 |
| *Morethia boulengeri* | 2 | m | 10 | Fox | T | 0 | 3 | 23.25 |
| *Morethia boulengeri* | 3 | f | 13 | Cat | C | 0 | 3 | 23 |
| *Morethia boulengeri* | 3 | f | 13 | Cat | T | 0 | 3 | 23 |
| *Morethia boulengeri* | 3 | f | 14 | Cat | C | 0 | 3 | 23 |
| *Morethia boulengeri* | 3 | f | 14 | Cat | T | 0 | 3 | 23 |
| *Morethia boulengeri* | 3 | f | 15 | Quoll | C | 0 | 3 | 23.25 |
| *Morethia boulengeri* | 3 | f | 15 | Quoll | T | 0 | 3 | 23.25 |
| *Morethia boulengeri* | 4 | f | 18 | Kangaroo | C | 0 | 3 | 23 |
| *Morethia boulengeri* | 4 | f | 18 | Kangaroo | T | 0 | 3 | 23 |
| *Morethia boulengeri* | 4 | f | 19 | Kangaroo | C | 0 | 3 | 23 |
| *Morethia boulengeri* | 4 | f | 19 | Kangaroo | T | 0 | 3 | 23 |
| *Morethia boulengeri* | 4 | f | 20 | Snake | C | 0 | 3 | 23.25 |
| *Morethia boulengeri* | 4 | f | 20 | Snake | T | 0 | 3 | 23.25 |
| *Morethia boulengeri* | 5 | m | 24 | Snake | C | 0 | 3 | 23 |
| *Morethia boulengeri* | 5 | m | 24 | Snake | T | 0 | 3 | 23 |
| *Morethia boulengeri* | 5 | m | 25 | Kangaroo | C | 0 | 3 | 23.25 |
| *Morethia boulengeri* | 5 | m | 25 | Kangaroo | T | 2 | 1 | 23.25 |
| *Morethia boulengeri* | 6 | f | 29 | Dingo | C | 2 | 1 | 22.25 |
| *Morethia boulengeri* | 6 | f | 29 | Dingo | T | 0 | 3 | 22.25 |
| *Morethia boulengeri* | 6 | f | 30 | Cat | C | 2 | 1 | 23.25 |
| *Morethia boulengeri* | 6 | f | 30 | Cat | T | 0 | 3 | 23.25 |
| *Morethia boulengeri* | 7 | f | 33 | Snake | C | 1 | 2 | 23 |
| *Morethia boulengeri* | 7 | f | 33 | Snake | T | 1 | 2 | 23 |
| *Morethia boulengeri* | 7 | f | 34 | Dingo | C | 2 | 1 | 23.25 |
| *Morethia boulengeri* | 7 | f | 34 | Dingo | T | 1 | 2 | 23.25 |
| *Morethia boulengeri* | 7 | f | 35 | Cat | C | 1 | 2 | 23.75 |
| *Morethia boulengeri* | 7 | f | 35 | Cat | T | 0 | 3 | 23.75 |
| *Morethia boulengeri* | 8 | f | 39 | Quoll | C | 3 | 0 | 23 |
| *Morethia boulengeri* | 8 | f | 39 | Quoll | T | 0 | 3 | 23 |
| *Morethia boulengeri* | 8 | f | 40 | Cat | C | 1 | 2 | 23.25 |
| *Morethia boulengeri* | 8 | f | 40 | Cat | T | 2 | 1 | 23.25 |
| *Morethia boulengeri* | 8 | f | 41 | Fox | C | 1 | 2 | 23.75 |
| *Morethia boulengeri* | 8 | f | 41 | Fox | T | 0 | 3 | 23.75 |
| *Morethia boulengeri* | 9 | f | 45 | Fox | C | 1 | 2 | 23 |
| *Morethia boulengeri* | 9 | f | 45 | Fox | T | 0 | 3 | 23 |
| *Morethia boulengeri* | 9 | f | 46 | Snake | C | 0 | 3 | 23.25 |
| *Morethia boulengeri* | 9 | f | 46 | Snake | T | 0 | 3 | 23.25 |
| *Morethia boulengeri* | 9 | f | 47 | Kangaroo | C | 0 | 3 | 23.75 |
| *Morethia boulengeri* | 9 | f | 47 | Kangaroo | T | 0 | 3 | 23.75 |
| *Morethia boulengeri* | 9 | f | 51 | Kangaroo | C | 0 | 3 | 23 |
| *Morethia boulengeri* | 9 | f | 51 | Kangaroo | T | 0 | 3 | 23 |
| *Morethia boulengeri* | 10 | f | 52 | Snake | C | 0 | 3 | 23.75 |
| *Morethia boulengeri* | 10 | f | 52 | Snake | T | 0 | 3 | 23.75 |
| *Morethia boulengeri* | 11 | f | 54 | Dingo | T | 0 | 3 | 23.25 |
| *Morethia boulengeri* | 11 | f | 55 | Dingo | C | 0 | 3 | 23 |
| *Morethia boulengeri* | 11 | f | 55 | Dingo | T | 0 | 3 | 23 |
| *Morethia boulengeri* | 11 | f | 56 | Fox | C | 0 | 3 | 23.25 |
| *Morethia boulengeri* | 11 | f | 56 | Fox | T | 0 | 3 | 23.25 |
| *Morethia boulengeri* | 11 | f | 57 | Dingo | C | 1 | 2 | 23.75 |
| *Morethia boulengeri* | 11 | f | 57 | Dingo | T | 0 | 3 | 23.75 |
| *Morethia boulengeri* | 12 | m | 61 | Quoll | C | 0 | 3 | 23 |
| *Morethia boulengeri* | 12 | m | 61 | Quoll | T | 0 | 3 | 23 |
| *Morethia boulengeri* | 12 | m | 62 | Kangaroo | C | 1 | 2 | 23.25 |
| *Morethia boulengeri* | 12 | m | 62 | Kangaroo | T | 0 | 3 | 23.25 |
| *Morethia boulengeri* | 12 | f | 65 | Cat | C | 0 | 3 | 23.25 |
| *Morethia boulengeri* | 12 | f | 65 | Cat | T | 0 | 3 | 23.25 |
| *Morethia boulengeri* | 12 | f | 66 | Cat | C | 0 | 3 | 23.5 |
| *Morethia boulengeri* | 12 | f | 66 | Cat | T | 0 | 3 | 23.5 |
| *Morethia boulengeri* | 12 | f | 67 | Quoll | C | 1 | 2 | 23.75 |
| *Morethia boulengeri* | 12 | f | 67 | Quoll | T | 0 | 3 | 23.75 |
| *Morethia boulengeri* | 13 | m | 71 | Kangaroo | C | 0 | 3 | 23.25 |
| *Morethia boulengeri* | 13 | m | 71 | Kangaroo | T | 0 | 3 | 23.25 |
| *Morethia boulengeri* | 13 | m | 72 | Quoll | C | 0 | 3 | 23.5 |
| *Morethia boulengeri* | 13 | m | 72 | Quoll | T | 0 | 3 | 23.5 |
| *Morethia boulengeri* | 13 | m | 73 | Snake | C | 0 | 3 | 23.75 |
| *Morethia boulengeri* | 13 | m | 73 | Snake | T | 0 | 3 | 23.75 |
| *Morethia boulengeri* | 14 | m | 76 | Cat | C | 0 | 3 | 23.25 |
| *Morethia boulengeri* | 14 | m | 76 | Cat | T | 0 | 3 | 23.25 |
| *Morethia boulengeri* | 14 | m | 77 | Dingo | C | 0 | 3 | 23.5 |
| *Morethia boulengeri* | 14 | m | 77 | Dingo | T | 0 | 3 | 23.5 |
| *Morethia boulengeri* | 14 | m | 78 | Quoll | C | 0 | 3 | 23.75 |
| *Morethia boulengeri* | 14 | m | 78 | Quoll | T | 0 | 3 | 23.75 |
| *Morethia boulengeri* | 15 | m | 82 | Snake | C | 1 | 2 | 23.25 |
| *Morethia boulengeri* | 15 | m | 82 | Snake | T | 1 | 2 | 23.25 |
| *Morethia boulengeri* | 15 | m | 83 | Kangaroo | C | 1 | 2 | 23.75 |
| *Morethia boulengeri* | 15 | m | 83 | Kangaroo | T | 0 | 3 | 23.75 |
| *Morethia boulengeri* | 16 | m | 87 | Fox | C | 0 | 3 | 23.5 |
| *Morethia boulengeri* | 16 | m | 87 | Fox | T | 0 | 3 | 23.5 |
| *Morethia boulengeri* | 16 | m | 88 | Fox | C | 2 | 1 | 23.75 |
| *Morethia boulengeri* | 16 | m | 88 | Fox | T | 0 | 3 | 23.75 |
| *Morethia boulengeri* | 17 | f | 92 | Fox | C | 1 | 2 | 23.25 |
| *Morethia boulengeri* | 17 | f | 92 | Fox | T | 0 | 3 | 23.25 |
| *Morethia boulengeri* | 17 | f | 93 | Kangaroo | C | 0 | 3 | 23.5 |
| *Morethia boulengeri* | 17 | f | 93 | Kangaroo | T | 0 | 3 | 23.5 |
